# A structured curriculum supporting biomedical trainees’ transition into independent academic positions and early career success

**DOI:** 10.1186/s12909-024-05370-w

**Published:** 2024-04-08

**Authors:** Mabel Perez-Oquendo, Gabriele Romano, David P. Farris, Varsha Gandhi, Ignacio I. Wistuba, Robert E. Tillman, Ryan Udan, Paolo Mangahas, Rama Soundararajan

**Affiliations:** 1 Department of Research, Education and Training, The University of Texas MD Anderson Cancer Center, Houston, TX 77030 USA; 2 Department of Pharmacology & Physiology, Drexel University College of Medicine, Philadelphia, PA 19102 USA; 3 Department of Translational Molecular Pathology, The University of Texas MD Anderson Cancer Center, Houston, TX 77030 USA; 4 Research Medical Library, The University of Texas MD Anderson Cancer Center, Houston, TX 77030 USA; 5 Department of Experimental Therapeutics, The University of Texas MD Anderson Cancer Center, Houston, TX 77030 USA; 6 Department of Education, Innovation and Technology, Baylor College of Medicine, Houston, TX 77030 USA; 7 Present address: Graduate School of Biomedical Sciences, Baylor College of Medicine, Houston, TX 77030 USA

**Keywords:** Academic careers in biomedical research, Postdoctoral fellows, Faculty-track soft-skills curriculum, Professional development skills, Scientists-in-training

## Abstract

The United States government makes a substantial investment in biomedical training programs each year. However, for most trainees, these opportunities do not translate into career progression in academic research pathways. Only about one-fifth of postdoctoral fellows eventually secure a tenure-track faculty position, and even among these candidates, attrition is high. Although a number of factors govern career choices and career longevity, the transition from trainee to faculty is a challenging process and requires knowledge and skills that are not necessarily developed during a traditional university experience. Many postdoctoral fellows receive adequate training in research skills and scientific communication, but new faculty report not being sufficiently prepared for the job search process and for starting their labs. To address this critical training gap, the ITERT core (Interdisciplinary Translational Education and Research Training) and the Office of Postdoctoral Fellows at the University of Texas MD Anderson Cancer Center implemented a structured course for both postdoctoral fellows and senior PhD students to provide formalized training for successfully navigating academic positions in biomedical research. Here we report on the pilot *Navigating Academic Careers* course conducted in 2021–2022 for 30 PhD students and postdocs. The nine-module course was conducted over 13 weeks in 25.5 h instructional sessions. The key educational objectives included 1) navigating the job application and the interview/negotiation process, 2) hiring, leading, and mentoring lab personnel and program support staff, 3) project administration and financial stewardship, 4) managing time and work-life balance and 5) developing collaborations, branding, personalized niche, and networking. Survey-based analysis at the time of the course was used to capture the participants’ assessment of the course content, organization, and delivery, with a follow-up survey conducted approximately 2 years post-course (2024) to evaluate longer-term impacts of the training. Initial in-course assessment revealed that 89.9% of respondents found the scope and instructional content appropriate, and 91.1% found the course relevant and applicable to their career needs. Longer-term post-course evaluation indicated that 80% of respondents applied the learnings of the course, that 80% reported feeling more confident in navigating an academic job search, and that 66.6% continued to report agreement with the course preparing them for their current role/ongoing job search, with 46.7% already securing jobs in academic research, including as independent faculty. The outcomes of this pilot course suggest that integrating this into the broader postdoctoral training curriculum can enhance both the transition and early-career success of talented scientists-in-training into working professionals in biomedical careers, as faculty and science-trained staff.

**Supplementary Information:**

The online version contains supplementary material available at 10.1186/s12909-024-05370-w.

## Introduction

Postgraduate trainees interested in an academic career in biomedical research typically undergo 5 to 6 years of training before taking the next steps in their career path [1]. For many postdoctoral fellows, this preparatory period is marked by advanced training in a research discipline and is often accompanied by a number of challenges related to end-of-training career transitions. A significant challenge is a relative lack of faculty jobs to match the high postdoctoral interest in these positions. Surveys over the past decade report that approximately 60% of postdoctoral fellows expect to transition to a tenure-track academic position [2–5]. Even for those that do transition into a faculty position, the transition is challenging. The most recent Association of American Medical Schools Faculty Roster indicates a 10-year attrition rate from academic medicine of 41% for first-time PhD assistant professors in basic sciences in US medical colleges [6]. The challenging nature of a new faculty responsibility is also reflected in recent studies showing that significant percentages of new faculty realize the need for competencies beyond research discipline-specific knowledge and skills [7].

Addressing this critical training need among both doctoral and postdoctoral trainees has been a focus of various approaches and interventions on the part a number of academic, governmental and professional institutions over the past 20 years [8–10]. These have included such efforts as the Burroughs Wellcome Fund (BWF) and the Howard Hughes Medical Institute’s (HHMI) 2002 course in scientific management - and the resulting manual, *Making the Right Moves*, that when published in 2004, fostered greater awareness of the need for professional training that was nascent in a number of academic institutions [11]. Around the same time, the National Postdoctoral Association was formed out of *Science*’s NextWave Postdoc Network meeting in April 2002, and early on defined core competencies for postdoctoral training that include communication skills, professional skills, grant writing skills, leadership/management skills, in addition to research skills, discipline-specific knowledge and responsible conduct of research [12]. While more recent efforts such as the NIH BEST Awards and longer running programs at institutions show a continuation of these efforts with a focus on evaluating the impact of these programs on supporting effective career transitions broadly, there are still critical gaps in how we train our postdoctoral fellows and what we require of them when they enter the academic job market [13–15]. Some major sources for these gaps are the still limited requirements by government agencies, foundations, and sponsored research agreements, to provide professional and career training for postdoctoral fellows; the unique structure and dynamics of the postdoctoral relationship characterized by being funded through faculty-awarded research grants; being comprised of a large international population and overall unique demographics; a variable (and asynchronous) appointment process that affects the duration of appointment and the availability of training; and variability in quality of mentorship and access to knowledge and expertise [8].

In this report, we share the development and initial outcomes of a comprehensive course that provides postdoctoral fellows and senior graduate students with a spectrum of knowledge, skills and abilities (tacit/soft-skills) intended to support their transition into advanced academic careers. The course, titled *Navigating Academic Careers*, was designed to provide a fundamental framework for understanding academic positions in biomedical research and critical considerations for successfully navigating these tracks, including faculty careers as principal investigators (PIs). Based on in-course evaluation and post-course outcomes of the pilot course offered through the ITERT core (Interdisciplinary Translational Education and Research Training) and the Office of Postdoctoral Fellows at the University of Texas MD Anderson Cancer Center (MD Anderson), we believe that this provides a model curriculum that be adapted and implemented for a wide range of trainees in basic-, translational-, and clinical science paths to support trainee effectiveness in their academic job search and transition into advanced research-related careers.

## Methods

### Course objectives

The objectives and topics for the *Navigating Academic Careers* course were determined by (1) trainee career-needs assessment, (2) NPA recommendations for postdoc competencies [12, 16], and (3) faculty input. A key objective was also to create a flexible career-preparedness tool that would enable continued learning during the COVID-19 pandemic period and beyond. The course was designed to address a variety of critical tacit skills: (1) navigating the job application and the interview/negotiation process; (2) hiring, leading, and mentoring lab personnel and program support staff; (3) project administration and financial stewardship; (4) managing time and work-life balance; and (5) developing collaborations, branding, personalized niche and networking, while also simultaneously developing a career-long support team of mentors and sponsors.

### Course design and delivery

The course was designed and implemented during the COVID pandemic (2021–2022). While the ability to offer this career-preparedness tool as an online mode was a critical consideration, given the COVID restrictions and the rapidly changing landscape of education and training, it also provided a potentially scalable platform for course delivery. The Canvas learning management system was used to develop a flexible yet interactive online platform for course delivery. The course was structured as nine independent modules (Table 1, expanded curriculum details in Supplementary Table 1) comprising 25.5 instructional units/hours (IU), with dedicated instructors for each module. The 9 learning modules were distributed over 13 weeks, with a maximum of 3.5 IU per week. Modules consisted of lectures taught by faculty or content experts from MD Anderson and neighboring research institutions within the Texas Medical Center in the US, and covered an overview of requirements for advanced academic research careers, including tenure-track faculty positions, insights into the academic job search process, logistics of running an independent research program, and other tacit/soft-skills needed to succeed in these career paths (Table 1, expanded details in Supplementary Table 1). The course was conducted online in live video sessions (*via* Zoom). Sessions were also recorded to create a training resource archive for institutional trainees, faculty, and employers.

**Table 1:** *Navigating Academic Careers*: Curriculum overview and major themes

Module	Theme
1	Academic Positions: The breadth of position titles in academic biomedical research, job descriptions and promotion requirements within each title
2	Networking: Garnering peer- and tiered-support, gaining visibility
3	The Interview Process: Application components, process checks
4	Scientific Leadership and Laboratory Management: Fundamental components and processes for working in- or running a research laboratory
5	Leadership in Practice: Taking Charge of Your Career: People- and project-leadership
6	Effective Time Management and Work-Life Balance
7	Mentorship and Sponsorship: Building a career-long support team
8	Developing your Niche: Defining and carving a pathway to independence
9	Career Conversations: One-on-one with Distinguished Academicians (early career-, mid career- and established professionals)

A total of 28 instructors participated in the course, teaching in the format of a seminar, chalk talk, workshop, or small group satellite discussions. Course instructors were selected based on area of expertise, level of experience, career trajectory in academia, mentoring track record, and commitment to continued mentoring beyond this course. The academic rank of the instructors spanned from early career assistant professors to senior professors including department chairs, intentionally, with the aim of providing a full-depth representation of the academic experience. Most instructors were faculty – assistant-, associate- or full professors at research institutions within the Texas Medical Center, but a few were content-expert administrative leads and leadership practitioners.

### Course participants

Postdoctoral fellows were recruited as priority, with senior graduate students accepted as course capacity allowed. Participation was voluntary and based on the individual’s interest in pursuing a career in the academic track. Participants consisted of 30 trainees: 25 postdoctoral fellows and 5 senior doctoral students. Of the 25 postdoctoral fellows, 19 were supported either by an individual fellowship, career development award, or by an institutional training grant/training program during their tenure as a fellow at MD Anderson. Similarly, 2 out of the 5 graduate students reported receiving such support during their training time at MD Anderson.

### Course evaluation

Attendance for each lecture was captured based on the registration/attendance report that Zoom generates, and participants recording a minimum of 80% course attendance were awarded a career-preparedness course-completion certificate. Two types of surveys were administered to evaluate the utility, effectiveness, learning outcomes and longer-term impact of the course: (1) an in-course survey after each module, while participants were still enrolled in the course, and (2) a 2 year post-course survey. All surveys were conducted using REDCap (Research Electronic Data Capture).

Course evaluation was designed using the well-established Kirkpatrick Four-Level Training Evaluation Model [17, 18]. The in-course surveys were intended to address Kirkpatrick Level 1 (“Reaction”: *How was the course received by the participants?)* and partly Level 2 (“Learning”: *Did the participants learn the stated educational objectives?)* with participant responses collected in 3 sections:

- *Section 1* (learner-centered) contained 6 statements related to course content, educational objectives, participant engagement, overall reaction to the program, and career-trajectory relevance. Two of these 6 statements were designed to evaluate the objective of each class using a Likert scale response (Table 2). For the other 4 statements (below), participants indicated their level of agreement or disagreement with the overall content and relevance of the course:
  1. The scope and variety of content that the session covered were appropriate.
  2. This session was relevant and applicable to my needs.
  3. I was fully engaged during this session.
  4. I would recommend this session to my colleagues.
- *Section 2* (trainer-centered) contained six statements related to the course organization and delivery. Participants indicated their level of agreement or disagreement with the following statements:
  1. The platform/venue for this session was appropriate for this type of training.
  2. The session was well organized and executed effectively.
  3. The objectives of the session were clearly defined.
  4. The presenter spoke clearly and at a good pace.
  5. The presentation was engaging, stimulating, and allowed me to gain a clear understanding of the topic covered.
  6. The presenter encouraged the attendees to ask questions and/or participate.
- *Section 3* contained two open-ended comments and questions related to course clarifications and suggestions for improvement.
  1. Please enter any questions or comments for the speaker that were not addressed during the session.
  2. What recommendations do you have to improve the overall quality and relevance of this training?

**Table 2:** “In-course” evaluation of the *Navigating Academic Careers* course

Class statements	Likert scale response				
**MODULE 1**					
** 1. Academic Positions**	Strongly Agree %	Agree %	Neutral %	Disagree %	Strongly Disagree %
I understand the different career paths available within academia.	26.7	60.0	0	0	13.3
I understand the considerations for appointment, promotion, and grant of tenure as faculty.	33.3	40.0	13.3	0	13.3
**MODULE 2**					
** 2. Networking**	Strongly Agree %	Agree %	Neutral %	Disagree %	Strongly Disagree %
I understand the significance of getting to know my peers.	100.0	0	0	0	0
I see the importance of taking advantage of alumni networks, social media platforms, and professional associations and societies.	100.0	0	0	0	0
**MODULE 3**					
** 3. Preparing Application Materials for a Faculty Position**	Strongly Agree %	Agree %	Neutral %	Disagree %	Strongly Disagree %
I have a better understanding of how to effectively draft a cover letter and CV.	53.8	46.2	0	0	0
I have a better sense of how to draft a research and diversity statement.	76.9	15.4	7.7	0	0
** 4. Preparing for the Interview to a Faculty Position (Part 1)**	Strongly Agree %	Agree %	Neutral %	Disagree %	Strongly Disagree %
I understand the components of a chalk talk.	62.5	37.5	0	0	0
I have a better sense of how to entice employers to get back to me.	62.5	37.5	0	0	0
** 5. Preparing for the Interview to a Faculty Position (Part 2)**	Strongly Agree %	Agree %	Neutral %	Disagree %	Strongly Disagree %
I have a better sense of how to properly prepare for an interview to a faculty position.	71.4	28.6	0	0	0
I understand how to negotiate the job offer.	14.3	57.1	0	28.6	0
**MODULES 4 and 5**					
** 6. Leadership and Laboratory Management**	Strongly Agree %	Agree %	Neutral %	Disagree %	Strongly Disagree %
I have a better understanding of how to establish IACUC, IRB, and IBC protocols.	55.6	11.1	22.2	11.1	0
I understand the importance of record keeping and sharing resources and data.	55.6	22.2	22.2	0	0
** 7. Managing Laboratory Finances**	Strongly Agree %	Agree %	Neutral %	Disagree %	Strongly Disagree %
I have a better understanding of how to put together a grant.	50.0	50.0	0	0	0
I have a better understanding of how to build and manage a budget.	33.3	66.7	0	0	0
** 8. Effective Management of Multiple Projects (Part 1)**	Strongly Agree %	Agree %	Neutral %	Disagree %	Strongly Disagree %
I understand the importance of collaboration and delegation.	85.7	14.3	0	0	0
I have a better understanding of the multiple techniques available for tracking progress.	28.6	57.1	14.3	0	0
** 9. Effective Management of Multiple Projects (Part 2)**	Strongly Agree %	Agree %	Neutral %	Disagree %	Strongly Disagree %
I have a better understanding of how to work on multiple research projects simultaneously.	28.6	57.1	14.3	0	0
I have a better sense of how to work with collaborators and how to track research progress.	28.6	57.1	14.3	0	0
** 10. Recruiting and Staffing Basics (Part 1)**	Strongly Agree %	Agree %	Neutral %	Disagree %	Strongly Disagree %
I have a clear understanding of the recruiting process.	71.4	28.6	0	0	0
I have a better understanding of how to interview and evaluate applicants.	57.1	42.9	0	0	0
** 11. Recruiting and Staffing Basics (Part 2)**	Strongly Agree %	Agree %	Neutral %	Disagree %	Strongly Disagree %
I have a better understanding of how to screen applicants.	37.5	62.5	0	0	0
I have a better understanding about how to staff and manage a lab.	25.0	62.5	0	0	12.5
**MODULE 6**					
** 12. Time Management and Work-Life Balance**	Strongly Agree %	Agree %	Neutral %	Disagree %	Strongly Disagree %
I understand the multiple demands on academics’ time and how to carve out space for truly important but not necessarily urgent goals.	71.4	21.4	7.1	0	0
I have learned about practical and effective strategies for carving out time for my priorities including self-care.	57.1	42.9	0	0	0
**MODULE 7**					
** 13. Mentorship and Sponsorship (Part 1)**	Strongly Agree %	Agree %	Neutral %	Disagree %	Strongly Disagree %
I understand the significance of mentorship in driving career success.	78.6	21.4	0	0	0
I see how mentorship can make a difference, especially in the early stages of my career and/or in proposal development.	100.0	0	0	0	0
** 14. Mentorship and Sponsorship (Part 2)**	Strongly Agree %	Agree %	Neutral %	Disagree %	Strongly Disagree %
I understand the difference between sponsorship and mentorship in supporting career success.	81.8	18.2	0	0	0
I understand how to develop and maintain sponsorship and mentorship relationships that fit my career needs and interests.	54.5	27.3	18.2	0	0
**MODULE 8**					
** 15. Developing your Niche: A pathway to independence**	Strongly Agree %	Agree %	Neutral %	Disagree %	Strongly Disagree %
I understand the significance of actively planning my career and professional development and using the IDP as a tool for success.	75.0	25.0	0	0	0
I understand how to leverage mentorship and other professional relationships in building my own network and gaining visibility as an independent scientist.	75.0	25.0	0	0	0
**MODULE 9**					
** 16. Career Conversations, Part 1: Postdoc to Early Career Faculty Transitions: Landing the job and getting started**	Strongly Agree %	Agree %	Neutral %	Disagree %	Strongly Disagree %
I understand the significance of staying funded (sustainability) and recruiting the best talent.	100.0	0	0	0	0
I am aware of the support networks for new faculty.	71.4	0	28.6	0	0
** 17. Career Conversations, Part 2: Early- to Established Faculty Career Advancement**	Strongly Agree %	Agree %	Neutral %	Disagree %	Strongly Disagree %
I understand the significance of staying funded and recruiting the best talent.	50.0	50.0	0	0	0
I am aware of the support networks for faculty.	25.0	75.0	0	0	0

The 2 year post-course survey was intended to address Level 2 (“Learning”: *Did the participants learn the stated educational objectives?*), as well as Level 3 (“Behavior”: *Did the participants apply what they learned*?) and Level 4 (“Results”: *Larger impact of the program/outcomes*) of the Kirkpatrick Model. Participants indicated their level of agreement or disagreement with the following nine statements related to actionable steps taken, as well as longer-term impact on major learning outcomes of the modules, overall impact on confidence level and skills acquisition for an academic job search, and bearing on current roles:

1. I feel more confident in approaching my current role, or ongoing job search, as a result of participation in the course.
2. I have applied learnings from this course.
3. I have reached out to a mentor/sponsor after participating in this course.
4. I updated my CV or draft of a research statement for a faculty position as a result of participating in this course.
5. I applied the interviewing skills learned in my ongoing job search as a result of participating in this course.
6. I was better prepared for managing multiple projects after participating in this course.
7. I was better prepared for leading projects/teams after participating in this course.
8. The course prepared me for my current role (or for the ongoing job search [if applicable]).
9. I considered applying for a faculty position as a result of participating in this course.

## Results

### In-course evaluation

**Fig. 1:**
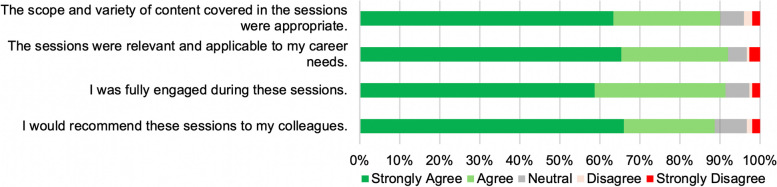
* Navigating Academic Careers*: overall course content and relevance evaluation metrics. Participants indicated their level of agreement or disagreement with four key statements related to overall scope and content of the course, as well their engagement during the course sessions and relevance to their career needs

Of the 30 postdoctoral fellows and senior graduate students who participated in the course, 73% (22/30) provided a complete *in-course* evaluation. The overall course content was analyzed with 4 statements using a Likert scale (Fig. 1). Overall, 89.9% of survey respondents concurred (agreed/strongly agreed) that the scope and variety of the course content were appropriate; 91.1% concurred that the sessions were relevant and applicable to their career needs; 91.4% fully engaged during the sessions; and 88.6% stated they would recommend the course to their colleagues, suggesting that the curriculum and educational objectives were well-crafted to address the specific training needs of the participants to understand and navigate academic positions in advanced biomedical research.

Participants also evaluated the content of each of the 17 included classes (covered over 9 modules) using 2 Likert scale questions per class, for a total of 34 class statements shown in Table 2. The classes evaluated included Academic positions; Networking for postdoctoral fellows, Preparing application materials for a faculty position; Preparing for the interview to a faculty position (Part 1 and 2); Leadership and laboratory management; Managing laboratory finances; Effective management of multiple projects (Part 1 and 2); Recruiting and staffing basics (Part 1 and 2); Time management and work-life balance; Mentorship (Part 1 and 2); Developing your niche: A pathway to independence; as well as Career Conversations Part 1: Postdoc to early career faculty-Landing the job and getting started, and Career Conversations Part 2: Early- to established faculty career advancement.

**Fig. 2:**
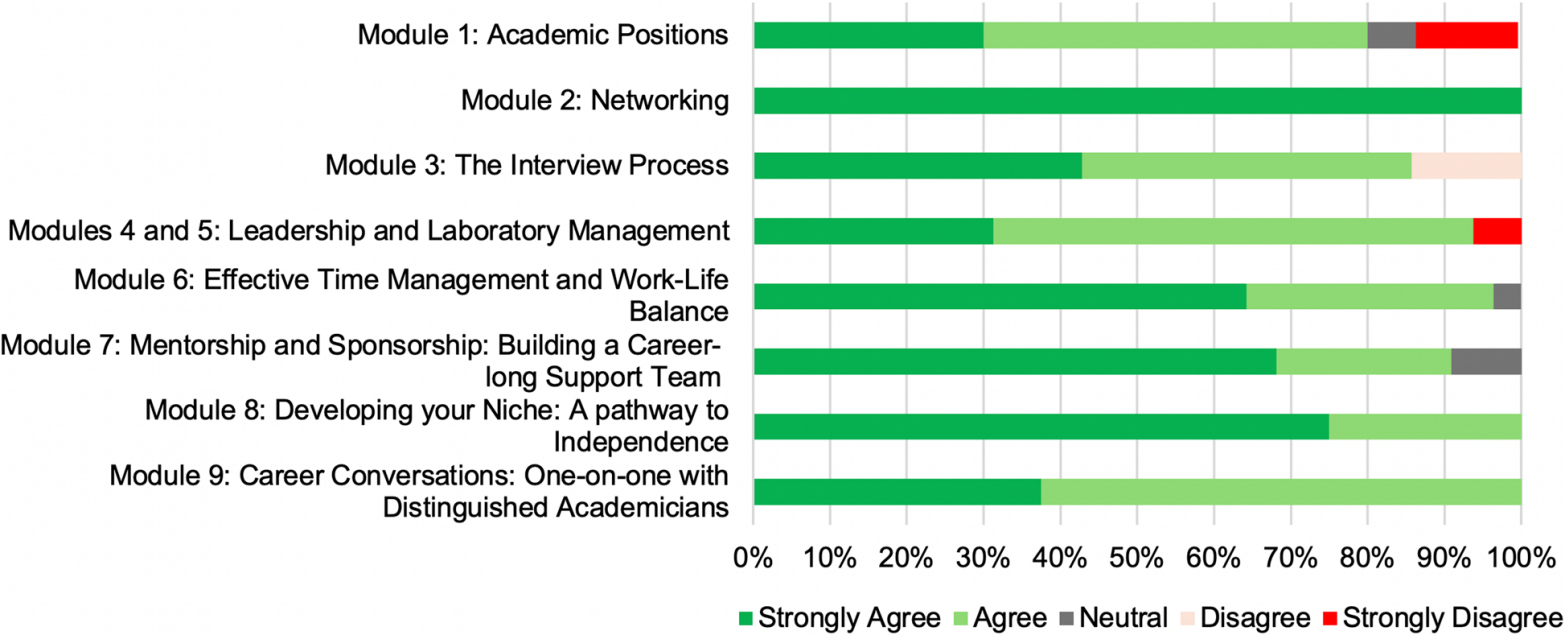
* Navigating Academic Careers*: educational objectives evaluation metrics. Participants indicated their level of agreement or disagreement with key statements designed to evaluate the educational objectives of each module in this course. Shown is the cumulative average scoring per learning module. Individual metrics are provided in Table 2

One-hundred percent of survey respondents unanimously concurred (agreed/strongly agreed) that this course had enabled them to (1) understand the significance of getting to know their peers; (2) see the importance of taking advantage of alumni networks, social media platforms, and professional associations/societies; (3) have a better understanding of how to draft effective CVs and cover letters; (4) understand the components of a chalk-talk, and prepare for an interview; (5) have a better understanding of the recruiting/staffing process, screen-, interview- and evaluate applicants for the team; (6) understand the significance of mentorship in driving career success and see how mentorship can make a difference, especially in the early stages of their career and/or in proposal development; (7) understand how mentorship and sponsorship can support career success; (8) understand the significance of actively planning for their career using individual development plans (IDPs), and leverage professional relationships in building out their network and gaining visibility as an independent scientist; (9) understand the significance of staying funded and recruiting the best talent; (10) better understand how to assemble an effective research grant proposal, build and manage a budget; (11) become aware of support networks for advanced faculty careers; (12) understand the importance of collaboration and delegation; and (13) learn practical strategies for prioritization both at work and also for self-care. Figure 2 shows the detailed metrics in Table 2, averaged *per* course module. 61.8% of the class statements (21/34) received a score of only “agree”/“strongly agree”, and 29.4% of the class statements (10/34) included “neutral” responses along with agreement (Table 2). Five out of 34 (14.7%) class statements included a combination of agreement and disagreement. Four of these 5 mixed-response questions had less than 14% disagreement. Seven of the 9 learning modules scored greater than 90% concurrence of agreement/strong agreement in meeting the educational objectives, with 3 of them scoring 100% concurrence (agreement/strong agreement) (Fig. 2). Taken together, our results strongly suggest that this course content enabled the participants to learn a variety of critical tacit/soft skills required for professional development and career advancement in biomedical research disciplines.

**Fig. 3:**
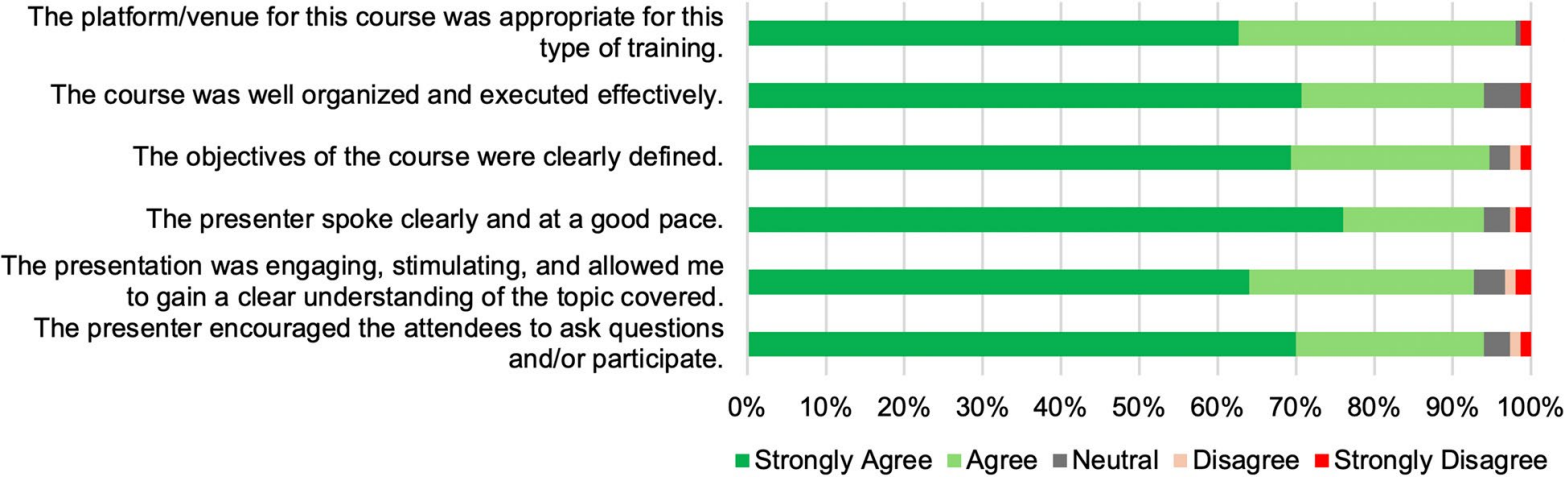
* Navigating Academic Careers*: course organization and delivery evaluation metrics. Participants indicated their level of agreement or disagreement with key indicators of the quality of course organization and delivery

To evaluate the course organization and delivery, participants were surveyed on 6 components using a Likert scale (Fig. 3). Ninety-eight percent of respondents concurred (agreed/strongly agreed) that the platform/venue (i.e., Zoom meeting/canvas) for the session was appropriate for this type of training. Further, 94% of respondents concurred that the session was well organized and executed effectively, while 94.6% concurred that the learning objectives of each session were clearly defined. Similarly, 94% of respondents concurred that the presenter spoke clearly and at a good pace, and 92.6% concurred that the presentation was engaging and stimulating allowing them to gain a clear understanding of the topic covered. Additionally, 94% concurred that the presenter encouraged them to interact and actively participate in the sessions. Overall, the course organization and delivery metrics received a high median score of 94.6%, confirming the success of both the online platform for delivery of such a career course and also the high quality of course organization and delivery by the instructors.

The majority of respondents who provided feedback about the course in free-response comments expressed the benefits of the course materials and also attested to the high-quality education from the instructors. Some also explicitly scripted the need for such a course while navigating the scholastic track in academia: “*I wish I had been able to attend something like this 2 years ago*”. Participants appreciated the welcoming and inclusive environment provided by the instructors, and highlighted the importance of such a course for a global training platform: “*Especially as an international trainee, it was great that the speaker pointed out the difference between a resume and a CV*” and “*Appreciate the tips given for candidates who do not have English as a first language. This is a key point that doesn’t get addressed often in sessions like this.*” For the Career Conversation: Landing the job and getting started (Part 1) class, trainees specifically appreciated the early-career role models: “*I appreciate the variety of experiences the external speakers were able to provide. Also, appreciate that they were all junior faculty-makes the conversation a little more relatable!*”

Participants also provided key recommendations to foster course discussion and further enhance the breadth of instruction. For the Time Management and Work-Life Balance class, the recommendation received was as follows: “*I would recommend having a longer session, and perhaps a group activity to discuss how to implement what we learned in our daily routine.*” For the class on Mentorship, one trainee opined: “*I would also perhaps include a slide or two about how to best build a solid relationship with your mentor(s), perhaps steps that are effective toward a fruitful mentor/mentee relationship*”. For the Academic Positions class, the suggestion received was as follows: “*Maybe one slide or more on the comparative RFA (Research Faculty Appointments-Non-Tenure Track) vs. non-RFA benefits.*” For the Preparing Application Materials for a Faculty Position class, multiple suggestions were received: “*It would have been great if the session were a bit longer. There was not that much time left to talk about the Diversity-Equity-Inclusion (DEI) statement*,” “*Seeing an example chalk talk would have been great; maybe a second session for that? or a longer session next time?*,” “*This topic could benefit from more time.*”

### Two-year post-course evaluation

**Fig. 4:**
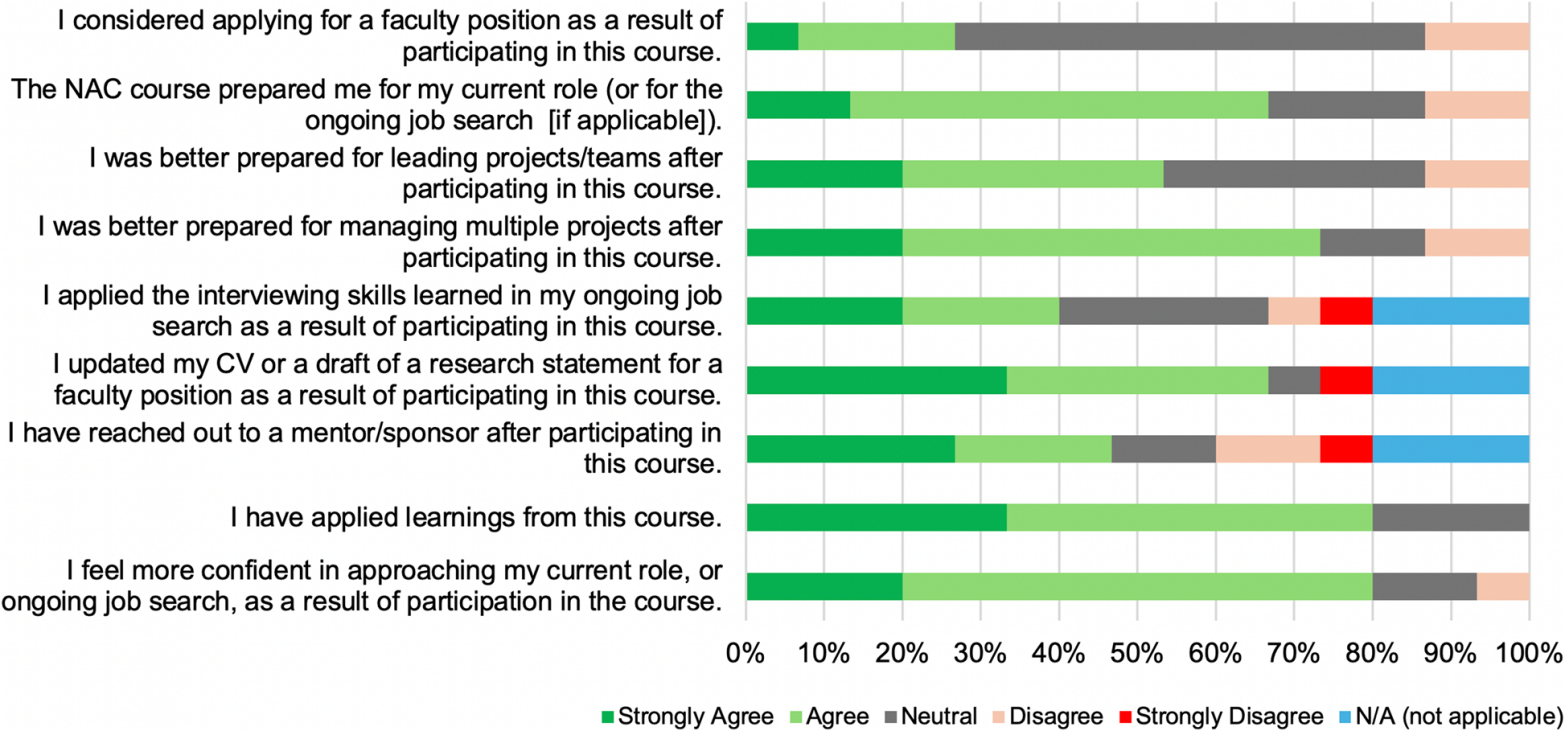
Longer-term impact of the *Navigating Academic Careers* course (two-year post-course evaluation metrics). Participants indicated their level of agreement or disagreement with key statements related to the longer-term career impact and confidence level in approaching their current roles/ongoing job search, as a result of participating in this career course

Of the 30 postdoctoral fellows and senior graduate students who participated in the course, 50% (15/30) provided a complete 2-year post-course follow-up evaluation. This evaluation was a 9-component assessment using a Likert scale, designed to address learning, as well as longer-term impact of the course learnings on career outcomes (Fig. 4). Eightly percent of respondents concurred (agreed/strongly agreed) that they applied the learnings from this course either in their current role or ongoing job search, and 66.7% concurred (agreed/strongly agreed) that they had updated their CV and research statement. Interestingly, while 80% of respondents concurred (agreed/strongly agreed) that they feel more confident in approaching their current role or ongoing job search directly as a result of participation in this course, only 26.7% decided to apply to a faculty position. Of the 15 post-course respondents, 46.7% (7/15) had secured advanced positions in academic research settings since taking this course, while 53.3% (8/15) are currently completing their ongoing postdoctoral fellowship (6/15) or PhD degree (2/15). Among the 7 trainees who successfully transitioned into an employed position in an academic institution: 1 is currently the director of a research office in an academic institution, 3 are assistant professors in research universities, 1 is an instructor in a research institution, 1 is a principal research scientist at a medical research institution, and 1 is a senior research scientist at a medical research institution. Further, 73.3% of respondents concurred (agreed/strongly agreed) that they were better prepared for managing multiple projects, and 66.6% concurred that they are better equipped even for the current role directly as a result of this course.

Taken together, our in-course and post-course evaluation metrics strongly endorse the short-term as well as longer-term professional development benefits of the *Navigating Academic Careers* course, in terms of supporting advanced academic careers in biomedical research.

## Discussion

In this work, we present data on impact and initial longitudinal outcomes of a pilot comprehensive career course designed to specifically aid trainees’ success in academic research, by providing structured instruction in a variety of tacit yet critical professional skills. The course, *Navigating Academic Careers*, was developed and piloted as part of a collaboration between the ITERT (Interdisciplinary Translational Education and Research Training) core and the Office of Postdoctoral Fellows at MD Anderson. Learning outcomes for the course were framed broadly within the 3 main objectives of ITERT: research education, career development, and supportive learning community. We intentionally structured this platform to provide an immersive experience on the tacit skills required to secure and manage careers in academia, particularly to serve the current generation of scientists. The instructional design of the modules allowed for workshops, break-out sessions, and self-learning components, serving multiple learner styles. While the course was delivered virtually due to the COVID-19 pandemic, the online delivery mode allowed flexibility, providing for both synchronous and asynchronous learning and was reported in evaluations as being effective. All live-streaming sessions were recorded, thus creating a permanent archive for prospective or enrolled trainees who could not attend a specific session. These resources generated during 2021–2022 continue to be routinely used to educate and train newly onboarding trainees at MD Anderson. Evaluations of the *Navigating Academic Careers* course support its role in preparing fellows for advanced research-focused careers (including faculty tenure-track careers), its role in instilling confidence in trainees for navigating academic career paths, as well as its inclusion as a valuable addition to current institutional programming.

While career offices at other institutions have variations of such training programs comprising one or more of the listed training elements, it is unclear if these resources are part of the routine curriculum for a postdoctoral fellowship, and if all graduating fellows necessarily experience the benefits equitably. Several highly respected institutions comparable to MD Anderson, such as Memorial Sloan Kettering Cancer Center, Dana-Farber Cancer Institute, and Johns Hopkins University School of Medicine offer seminars, workshops, retreats, and courses related to various leadership and career development topics, but none are formalized mandatory courses for completing a postdoctoral fellowship [19–21]. There are two global programs that offer training to postdocs and have a similar course structure to the *Navigating Academic Careers* course (below), but neither covers the breadth of content that our course does, nor is their content tailored specifically for graduate student/postdoctoral medical researchers interested in pursuing academic careers. The *Postdoc Academy for Transformational Leadership* is based in Europe and is available to early-career researchers working in sustainability at European research institutions. This course is comprised of four seminars held over two years, and each seminar is held at one of the four sponsoring academic centers. The primary difference is that modules cover topics related to sustainability leadership such as human-environment research, systems thinking, research methodology, and career development [22]. The *Postdoc Academy* is available as a Massive Open Online Course (MOOC) on the edX platform as well as a separate website (www.postdocacademy.org). It was developed broadly to meet the needs of postdoctoral researchers in all fields [23]. Postdoc Academy is structured in two online asynchronous courses: (1) Succeeding as a Postdoc and (2) Building Skills for a Successful Career. As with the *Navigating Academic Careers* course, the goals of *Postdoc Academy* align with the core competencies outlined by the National Postdoctoral Association. The second course offers modules that cover several of the same learning objectives and topics as the *Navigating Academic Careers* course; however, our course offers unique content such as Networking, Effective Time Management, Work-Life Balance, and Developing Your Niche (unique specialization/building your personal brand). Our modular platform allows postdoctoral fellows to fill this training gap and equip themselves with the necessary tools to face the job search process and launch their career as independent scientists in academia. Eighty percent of participants who responded to the follow-up survey two years after completing the course reported feeling more confident in approaching their current role, or ongoing job search, as a result of participation in the *Navigating Academic Careers* course. Although 53.3% of these participants are still in their training positions, the other 46.7% have advanced to positions such as director of a research office in an academic institution, assistant professors, instructor, principal research scientist, and senior research scientist.

The responses to the 2-year post-course survey also provided interesting data to further explore. Aspects of the course such as project management were reported as being advantageous to participants *regardless* of their current role and overall, participants applied the learnings from the course to their current role. Other outcomes queried such as applying interviewing skills or updating their CV as a result of the course were not just as prevalent - perhaps reflecting the fact that a number of participants are still in a training position and just have not yet had the opportunity for those activities. Finally, only 26.7% of respondents indicated that the course had an impact on them applying to a faculty position. While this is not surprising as the participants were selected based on their interest in an academic position, it would indicate the training can provide a level of further support and perhaps confidence for participants to pursue their academic career interests.

This course is framed in a context that has seen the delivery of education changing drastically due to a global pandemic. In a recent survey, 61% of postdoctoral fellows stated that the pandemic has negatively affected their career prospects, and another 25% stated that its cumulative effects on their career remain uncertain [24]. The combined synchronous and asynchronous online learning platform of our new course allows for a great degree of learning flexibility, and provides trainees the much-needed networking opportunities that were strongly impaired in the last few years due to the pandemic. Currently, there is a pressing need to embrace remote learning [25] and create futuristic learning modules to address the evolving landscape of education and training. The *Navigating Academic Careers* course was offered *via* Zoom, and 98% of the participants who completed the survey concurred (agreed/strongly agreed) that this platform/venue was appropriate for this type of training. Further, over 90% of the participants were in favor of the instructors’ presentation skills and ability to engage students in the learning-centered approach on this online platform. Hence, this course offered a mode to provide continued career-preparedness and online-easy access. This experience also equipped us with a potentially scalable career-preparedness tool for the future.

### Limitations and future directions

The work presented has some limitations. Primarily, this is a single institute study. The course was developed and delivered at the University of Texas MD Anderson Cancer Center, which is a non-profit institution located in the Texas Medical Center in the US. Further, we pooled results from participants with different training and cultural backgrounds. The evaluation time was also relatively short; participants had 2 h after the end of each class to complete the survey, the default time-setting in RedCap. Based on informal participant feedback, it was revealed that while the opportunity to provide a quick response within the 2-hour time-frame enabled first-impression feedback when the course was still fresh in their minds, a longer-term feedback window may have allowed more participants to respond, as well as allowed participants more time to reflect on the sessions, before providing feedback. In the future, course evaluation will benefit from the extended feedback collection time-frame, as well as extended multicultural postdoctoral representation of nearby research- and educational institutions within the Texas Medical Center, such as Baylor College of Medicine, The University of Texas Health Science Center, and Houston Methodist Research Institute, as well as other institutions across the US. Of the 30 trainees who participated in the institutional launch of this course, 21 indicated that during their tenure as a fellow at MD Anderson they were supported by an individual fellowship, career development award, or institutional training grant/training program. Some of the individual fellowships available to researchers also included a professional development training component. Therefore, for the next cohort, it will be important to assess the entry-level skills that all participants bring to the course and from there on, measure the success of the course in improving trainees’ career-preparedness. The structure and content of the course were presented at the 2021 TEACH-S educational symposium (Texas Educator’s Academies Collaborative for Health Professions – Southeast) to disseminate this concept to the greater scientific community and seek feedback from educators across multiple fields in biomedical sciences. The course was very well received by the educators, who also pointed out the necessity to include a distinct module on resilience and self-advocacy in future iterations. Although these concepts were constant themes highlighted and discussed in every module, we do agree that the formal integration of dedicated sessions on “building resilience” and “self-advocacy” into the course would strongly benefit the participants. Lastly, continued additional evaluations by our initial participant cohort over the next 5–10 years will highlight the course’s sustained success in preparing the next generation of scientists vested in academic careers.

## Conclusions

We piloted a comprehensive career development course designed to provide senior PhD students and postdoctoral trainees with a host of tacit skills required to successfully transition into and establish academic careers in biomedical research. The nine-module, online course, using learner-centered approaches for career preparedness served as a structured “one-stop shop” for skills to provide early-career scientists with critical skills and tools to approach academic careers with greater competency and confidence. Evaluation of the course during delivery, and a 2-year post-course evaluation provided strong preliminary evidence that the curriculum supports participants’ confidence level in approaching their current roles and/or transition to advanced jobs. While longitudinal evaluation of cohorts will continue to determine longer term outcomes, we believe that integrating this course into the broader postdoctoral training curriculum can enhance both the transition and early-career success of talented scientists-in-training into working professionals in biomedical careers, as faculty and science-trained staff.

### Supplementary Information

**Supplementary Material 1.**

**Supplementary Material 2.**

## Data Availability

The datasets used and/or analyzed during the current study are available from the corresponding author upon reasonable request for non-commercial academic purposes.

## Abbreviations

PI: Principal Investigator
MD Anderson: The University of Texas MD Anderson Cancer Center
REDCap: Research Electronic Data Capture
ITERT: Interdisciplinary Translational Education and Research Training Core
TEACH-S: Texas Educator’s Academies Collaborative for Health Professions – Southeast
