# Supplementary material for: A structured curriculum supporting biomedical trainees’ transition into independent academic positions and early career success
Source: BMC Med Educ. 2024 Apr 8;24:379. doi: 10.1186/s12909-024-05370-w (PMC11000405; doi:10.1186/s12909-024-05370-w)
Supplement: Supplementary file 1 — Supplementary Material 1. [file 12909_2024_5370_MOESM1_ESM.docx]

**Supplementary Table 1.** *Navigating Academic Careers*: Detailed Course Curriculum

| **Module 1: Academic Positions** | | |
| --- | --- | --- |
| Week 1 | 1.5 Hours | -Faculty and Non-Faculty tracks -MD Anderson, national and global - Titles, roles and job descriptions -Securing academic positions - Challenges and opportunities -Understanding common requirements for promotion and career advancement in an academic setting |
| **Module 2: Networking** | | |
| Week 4 | 2 hours | -Getting to know your peers -Promoting trainee science in the new age -Significance and role of social media -Professional associations/societies -LinkedIn basics -Leveraging “Alumni Networks” |
| **Module 3: The Interview Process** | | |
| Week 1 | 1 Hour (workshop) | -Drafting an effective CV and cover letter/s -Writing effective "Research Summary" statements -Making an impact - written and oral - ground rules for effective communication -The job talk -A chalk talk -The follow-up process -Negotiating an academic position: What are essential considerations? |
| Week 2 | 1 Hour (workshop) |  |
| Week 3 | 1 hour |  |
| **Module 4: Scientific Leadership and Laboratory Management** | | |
| Week 5 | 3.5 hours (including Small Group Study) | -Fundamentals of grantsmanship, Funding a lab -Budget essentials, Financial management -Recruiting/Staffing basics -Developing teaching skills and experience, Knowledge sharing -Protocols - Refresher \| IACUC, IRB, IBC: Why are they important? -MTAs and Research Collaboration Agreements: What are they? -Project management basics -Significance of periodic evaluations, Techniques for tracking progress -Sharing resources/data, Record keeping -Publications – Authorship 101 |
| Week 6 | 1.5 Hours |  |
| Week 7 | 2 hours (including Small Group Study) |  |
|  |  |  |
| **Module 5: Leadership in Practice: Taking Charge of Your Career** | | |
| Week 7 | 1.5 hours | **Sub-Module 1: Leading Teams (People Leadership)** -Influence building -Building sustainable teams, Inspiring trust -Recognizing talent, Enabling the best in all team members -Promoting a culture of inclusivity and mutual respect |
| Week 10 | 1.5 hours | **Sub-Module 2: Leading Projects (Project Leadership)** -Building and driving a unique career in science-Commitment -Establishing expectations -Steering a team to deliver shared goals: Impact of sound leadership on project success -Conflict resolution, Communication |
| **Module 6: Effective Time Management and Work-Life Balance** | | |
| Week 8 | 1.5 Hours | -Prioritization and Productivity -Goal setting -Securing and maintaining effective collaborations -Delegation -Work-life balance: Striking harmony |
| **Module 7: Developing Your Niche (unique specialization/building your personal brand)** | | |
| Week 9 | 2 hours | -Pathway to independence, building recognition for work distinct from PI -Leading team science: Ground rules -Visibility in the scientific network -Drafting a blueprint for success - "Individual Development Plans (IDPs)" |
| **Module 8: Mentorship and Sponsorship** | | |
| Week 11 | 2 hours | -Role of mentors and sponsors in promoting trainee careers -How do you find mentors and sponsors? -Assembling a (career-long) support team -Designing a project in your postdoc years to take forward as a faculty: Working with your mentor |
| **Module 9: Career Conversations: One-on-One with Distinguished Academic Researchers (Early Career-, Mid Career- and Established Researchers)** | | |
| Week 12 | 1.5 Hours | -How/Where do you begin your job search? -Your first (independent) research project as PI -Your first grant application as faculty -Staying funded as a new/established faculty and recruiting the best talent: Critical considerations -Retrospective lessons: Support networks for new and established faculty |
| Week 13 | 2 hours |  |
