# Supplementary material for: A structured curriculum supporting biomedical trainees’ transition into independent academic positions and early career success
Source: BMC Med Educ. 2024 Apr 8;24:379. doi: 10.1186/s12909-024-05370-w (PMC11000405; doi:10.1186/s12909-024-05370-w)
Supplement: Supplementary file 2 — Supplementary Material 2. [file 12909_2024_5370_MOESM2_ESM.docx]

**Supplementary Table 2.** *Navigating Academic Careers:* Course Instructors

| **Name** | **Title** | **Department/Institution** | **Current position and affiliation (if changed since teaching in the course)** |
| --- | --- | --- | --- |
| Dr. Danna Kurtin | Associate Vice President | Faculty Academic Affairs, UT MD Anderson Cancer Center, Houston, TX |  |
| Ms. Janice Simon | Leadership Practitioner | The Leadership Institute, UT MD Anderson Cancer Center, Houston, TX |  |
| Dr. John Heymach | Chair, Professor | Thoracic/Head and Neck Medical Oncology, UT MD Anderson Cancer Center, Houston, TX |  |
| Dr. Jeffrey Rosen | Distinguished Service Professor | Molecular & Cellular Biology, Baylor College of Medicine, Houston, TX |  |
| Dr. Hoda Badr | Associate Professor | Epidemiology & Population Sciences, Baylor College of Medicine, Houston, TX | Professor, Epidemiology & Population Sciences, Baylor College of Medicine, Houston, TX |
| Dr. Sina Safayi | Director | Career Development and Industry Outreach, Rush University Medical Center, Chicago, IL | Principal Scientist - Preclinical Research, Johnson & Johnson MedTech, Cincinnati, OH |
| Dr. Chandra Bartholomeusz | Associate Professor | Breast Medical Oncology, UT MD Anderson Cancer Center, Houston, TX |  |
| Dr. Nidhi Sahni | Assistant Professor | Epigenetics & Molecular Carcinogenesis, UT MD Anderson Cancer Center, Houston, TX | Associate Professor, Epigenetics & Molecular Carcinogenesis, UT MD Anderson Cancer Center, Houston, TX |
| Dr. Keri Schadler | Assistant Professor | Pediatrics - Research, UT MD Anderson Cancer Center, Houston, TX |  |
| Dr. George Calin | Professor | Translational Molecular Pathology, UT MD Anderson Cancer Center, Houston, TX |  |
| Dr. Lawrence Kwong | Associate Professor | Translational Molecular Pathology, UT MD Anderson Cancer Center, Houston, TX |  |
| Dr. Lauren Byers | Associate Professor | Thoracic/Head and Neck Medical Oncology, UT MD Anderson Cancer Center, Houston, TX | Professor, Thoracic/Head and Neck Medical Oncology, UT MD Anderson Cancer Center, Houston, TX |
| Dr. Subrata Sen | Professor | Translational Molecular Pathology, UT MD Anderson Cancer Center, Houston, TX |  |
| Dr. Sue-Hwa Lin | Professor | Translational Molecular Pathology, UT MD Anderson Cancer Center, Houston, TX |  |
| Dr. Celia Garcia-Prieto | Associate Director, Research Planning & Development | UT MD Anderson Cancer Center, Houston, TX | Department Administrator, Translational Molecular Pathology, UT MD Anderson Cancer Center, Houston, TX |
| Dr. Rama Soundararajan | Associate Professor | Translational Molecular Pathology, UT MD Anderson Cancer Center, Houston, TX |  |
| Dr. Varsha Gandhi | Professor | Experimental Therapeutics, UT MD Anderson Cancer Center, Houston, TX |  |
| Dr. Ronald DePinho | Professor | Cancer Biology, UT MD Anderson Cancer Center, Houston, TX |  |
| Dr. Robert Tillman | Director of Faculty Development | Faculty Affairs and Faculty Development, Baylor College of Medicine, Houston, TX | Department of Education, Innovation and Technology, Baylor College of Medicine, Houston, TX 77030, USA. |
| Dr. Eduardo Vilar-Sanchez | Associate Professor | Clinical Cancer Prevention, UT MD Anderson Cancer Center, Houston, TX | Department Chair *ad interim*, Professor, Clinical Cancer Prevention, UT MD Anderson Cancer Center, Houston, TX |
| Dr. Krishna Bhat | Associate Professor | Translational Molecular Pathology, UT MD Anderson Cancer Center, Houston, TX | Associate Professor, Mayo Clinic, Phoenix, AZ |
| Dr. Vito Rebecca | Assistant Professor | Biochemistry and Molecular Biology, Johns Hopkins Bloomberg School of Public Health, Baltimore, MD |  |
| Dr. Florian Karreth | Assistant Member | Molecular Oncology and Cutaneous Oncology, Moffitt Cancer Center, Tampa, FL | Associate Member, Molecular Oncology and Cutaneous Oncology, Moffitt Cancer Center, Tampa, FL |
| Dr. Edward Hartsough | Assistant Professor | Pharmacology & Physiology, Drexel University College of Medicine, Philadelphia, PA |  |
| Dr. Francesca Taraballi | Assistant Professor | Orthopedic Surgery, Academic Institute, Houston Methodist, Houston, TX |  |
| Dr. Sendurai Mani | Professor | Translational Molecular Pathology, UT MD Anderson Cancer Center, Houston, TX | Professor and Associate Director, Translational Oncology, Brown University Legorreta Cancer Center, Providence, RI |
| Dr. Marie Webster | Resident Faculty, Assistant Professor | The Lankenau Institute for Medical Research, Wynnewood, PA |  |
